# Identification of an Epoxide Metabolite of Lycopene in Human Plasma Using ^13^C-Labeling and QTOF-MS

**DOI:** 10.3390/metabo8010024

**Published:** 2018-03-20

**Authors:** Morgan J. Cichon, Nancy E. Moran, Ken M. Riedl, Steven J. Schwartz, Steven K. Clinton

**Affiliations:** 1 Department of Food Science & Technology, The Ohio State University, Columbus, OH 43210, USA; riedl.7@osu.edu (K.M.R.); schwartz.177@osu.edu (S.J.S.); 2 The Ohio State Comprehensive Cancer Center, Columbus, OH 43210, USA; nancy.moran@bcm.edu (N.E.M.); steven.clinton@osumc.edu (S.K.C.); 3 Division of Medical Oncology, Department of Internal Medicine, The Ohio State University Comprehensive Cancer Center, Columbus, OH 43210, USA

**Keywords:** lycopene, metabolite, mass spectrometry, stable isotopes

## Abstract

The carotenoid lycopene is a bioactive component of tomatoes and is hypothesized to reduce risk of several chronic diseases, such as prostate cancer. The metabolism of lycopene is only beginning to be understood and some studies suggest that metabolites of lycopene may be partially responsible for bioactivity associated with the parent compound. The detection and characterization of these compounds in vivo is an important step in understanding lycopene bioactivity. The metabolism of lycopene likely involves both chemical and enzymatic oxidation. While numerous lycopene metabolites have been proposed, few have actually been identified in vivo following lycopene intake. Here, LC-QTOF-MS was used along with ^13^C-labeling to investigate the post-prandial oxidative metabolism of lycopene in human plasma. Previously reported aldehyde cleavage products were not detected, but a lycopene 1,2-epoxide was identified as a new candidate oxidative metabolite.

## 1. Introduction

Epidemiological studies have demonstrated an association between the consumption of tomato products and the carotenoid lycopene and a decreased risk of prostate cancer [1,2], a relationship supported by experimental models [3,4]. Lycopene is the pigment responsible for the red color of tomatoes and is one of the predominant phytochemicals in the tomato [5]. Lycopene metabolism is believed to progress through chemical or enzymatic oxidative cleavage of the hydrocarbon chain. Provitamin A carotenoids are centrally and oxidatively cleaved to generate vitamin A metabolites that interact with specific retinoid receptors to impact cell biology, while eccentric cleavage is likely an initial step in degradation pathways [6]. The mammalian genes coding for central and eccentric carotenoid cleavage have been characterized, and it is proposed that oxidative cleavage metabolites of lycopene may be responsible for some of the biological effects associated with lycopene [7], perhaps acting as partial agonists or antagonists to receptors in the steroid receptor superfamily [3,8,9]. Potential oxidation products (apo-lycopenoids) have been identified in vitro and include aldehydes, ketones, and acids [10,11]. Our understanding of lycopene metabolism is far more limited than for provitamin A carotenoids, and only a few potential lycopene metabolites have been reported in vivo. Our group has previously observed several apo-lycopenals (apo-6′-, apo-8′-, apo-10′-, apo-12′, and apo-14′-lycopenal) in human plasma [12]. These compounds are also found in small quantities in lycopene-containing foods, including raw tomatoes, tomato sauce, and tomato juice. Thus, it is unclear whether they are absorbed from the diet, are products of in vivo chemical or enzymatic metabolism, or perhaps are the result of both mechanisms.

Isotopic labeling has been used as a strategy for investigating carotenoid absorption, metabolism, and excretion in vivo. ^14^C-labeling was used to study the biodistribution of lycopene in rodents [13,14] and to investigate the long-term bioavailability of lycopene in human plasma from a single oral dose [15]. Radiolabeling is a highly sensitive technique, but as ^14^C is radioactive, the amount of ^14^C-lycopene that can be safely administered is limited and cannot be used to assess carotenoid metabolism at concentrations found in the diet. For this reason, it is difficult to obtain concrete structural information for lycopene metabolites using ^14^C-labeling. Stable isotope labeling provides an alternative low-risk approach, where ^13^C-labeled carotenoids can be consumed at concentrations found in the diet. This strategy has been used to study β-carotene absorption and conversion to vitamin A [16,17], as well as lycopene [18] and phytoene [19] pharmacokinetics.

The objective of this study is to investigate the oxidative metabolism of lycopene in humans using a ^13^C-label. Plasma samples for this analysis were obtained from a recent study conducted by our group [18]. We expect labeling with a stable isotope will offer enhanced sensitivity to identify biological metabolites using liquid chromatography-quadrupole time-of-flight mass spectrometry (LC-QTOF-MS).

## 2. Results 

### 2.1. Targeted Search for Apo-Lycopenals

Given our group’s previous identification of apo-lycopenals in human plasma, we expected to observe ^13^C-labeled aldehyde cleavage products of lycopene in the plasma of subjects from this study. The LC-MS method used for the plasma analysis was, therefore, optimized to detect the various apo-lycopenals expected (apo-6′-, apo-8′-, apo-10′-, apo-12′-, and apo-14′-lycopenal). This study provided a dose of 10.2 mg ^13^C-lycopene, which is physiologically relevant. However, masses corresponding to the ^13^C-labeled apo-lycopenals were not detected in the plasma of the eight subjects at the any of the time points examined. Additionally, native, unlabeled apo-lycopenals were also not detected. 

### 2.2. Identification of New Lycopene Metabolites

**Figure 1:**
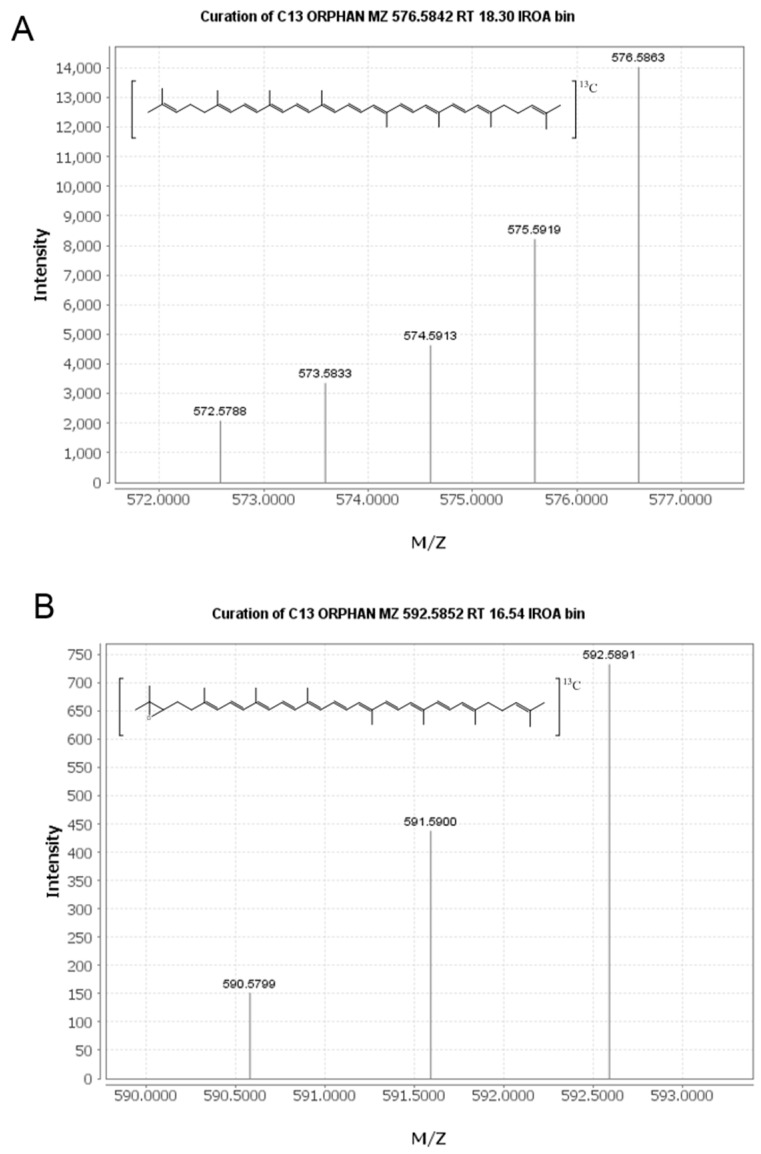
Mass spectra of the ^13^C-labeled lycopene (**A**) and lycopene epoxide (**B**) detected with the IROA ClusterFinder software.

An untargeted approach was taken to mine the full-scan LC-MS data collected and identify new plasma metabolites of lycopene. The IROA ClusterFinder software (version 1, build 81) was used to search the data and extract mass spectral features with isotopic distributions characteristic of the ^13^C enrichment provided in the tracer dose. This approach was validated with the detection of ^13^C-lycopene in our plasma samples (Figure 1A). All features detected by the software were manually reviewed to eliminate false positives resulting from noise in the spectra. For a feature to be considered a labeled metabolite, it needed to be clearly present post-dose, but not at baseline.

**Figure 2:**
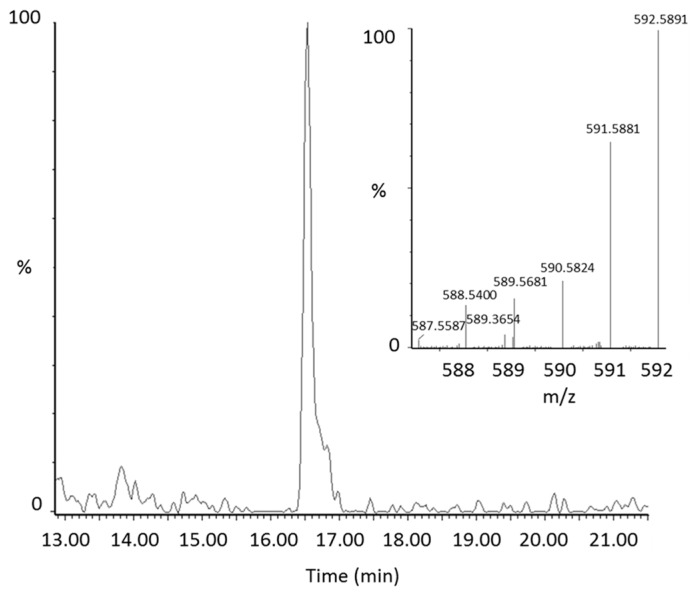
Representative extracted ion chromatogram of the lycopene epoxide (*m*/*z* 592.58) in plasma with the corresponding mass spectrum of the peak.

From manual review of the ClusterFinder data, we discovered another labeled compound besides ^13^C-lycopene that was present post-dose, but not at baseline and had the same characteristic reverse isotope distribution as the labeled lycopene (Figure 1B). With an *m*/*z* of 592.58, this compound was determined to be a ^13^C-labeled lycopene epoxide as the mass corresponds to the addition of oxygen to the parent molecule. The compound had a retention time of 16.5 min (Figure 2) and eluted in the same region of the chromatogram as all-*trans*-lycopene (18.2 min), 5-*cis*-lycopene (18.4 min), and other lycopene *cis* isomers (14.8–16.8 min), demonstrating structural similarity. 

**Figure 3:**
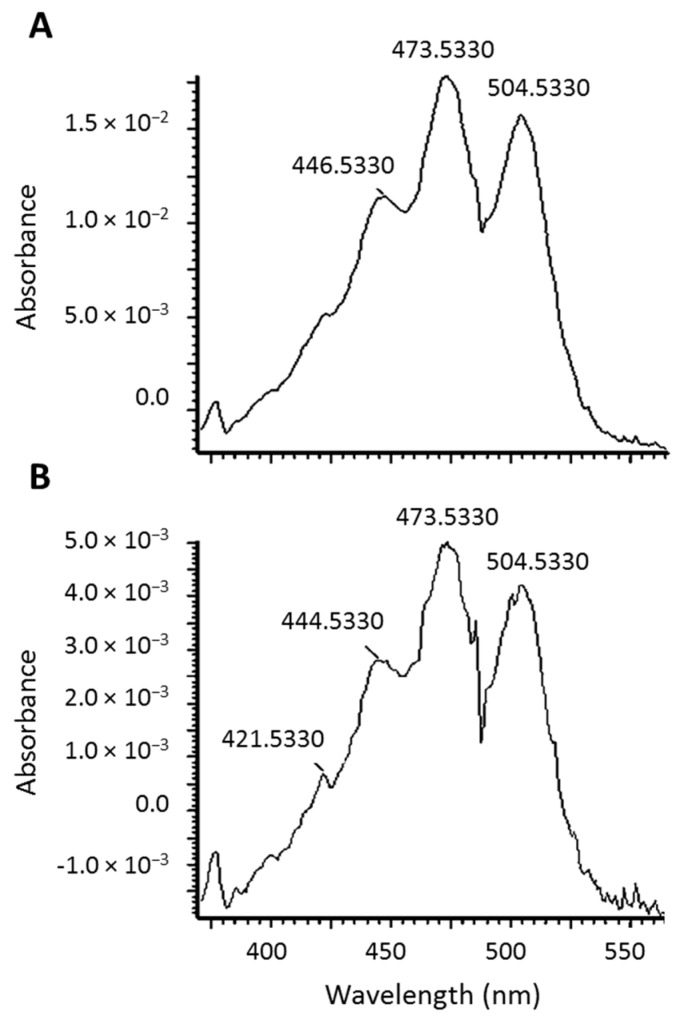
UV-Vis spectra of *all-trans*-lycopene (**A**) and the lycopene epoxide (**B**).

Lycopene mono- and di-epoxides have been reported in tomato products, along with NMR and UV-Vis confirmation [20]. Carotenoids have characteristic absorption spectra and the epoxide observed here was found to have a similar absorption spectrum to all-*trans*-lycopene (Figure 3). Therefore, we hypothesize that epoxidation is occurring at the 1,2 position (see structure in Figure 1B) where the chromophore and UV-Vis spectrum would be unaltered [21]. This is in agreement with the UV-Vis data reported previously for lycopene 1,2-epoxide [20]. Epoxidation of lycopene at the 5,6 position has also been reported, but this alteration causes a shortening of the chromophore and a hypsochromic shift of approximately 20 nm [22,23]. The terminal double bond on the lycopene structure is the only position where epoxidation would not alter the absorption spectrum.

### 2.3. Quantitation of ^13^C-lycopene 1,2-Epoxide in Plasma

**Figure 4:**
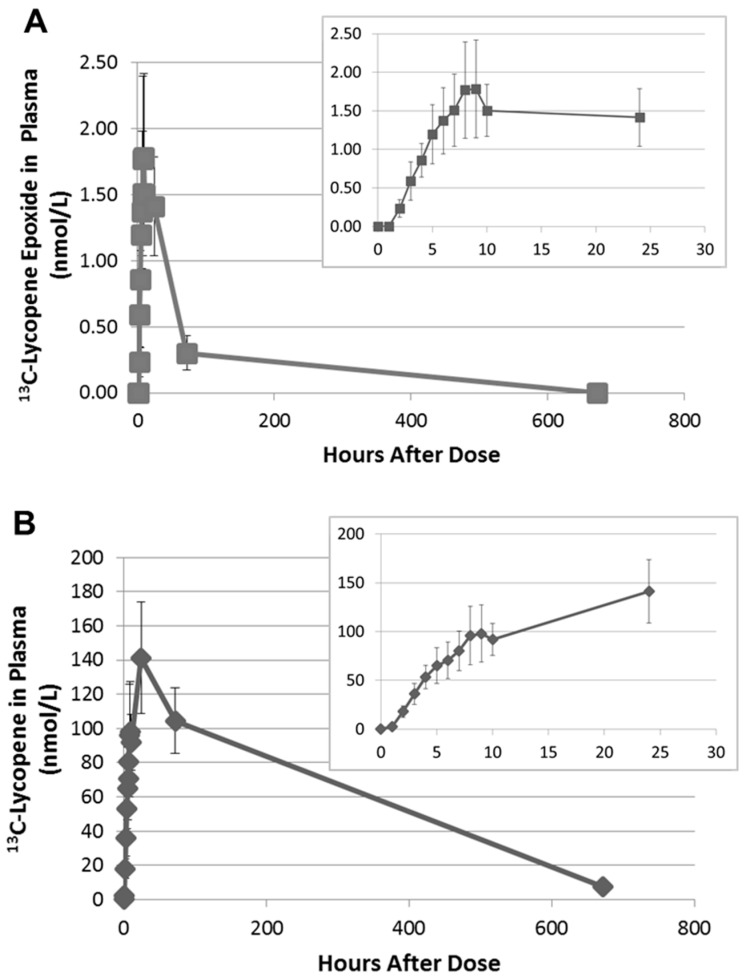
Average appearance of the ^13^C-lycopene epoxide metabolite (**A**) and the ^13^C-lycopene parent (**B**) in the plasma of subjects (±SEM) with insets zoomed to the first 24 h. (^13^C-lycopene data have been previously published [18] and are visualized here for comparison purposes.).

Upon further inspection of the data, the ^13^C-labeled lycopene 1,2-epoxide was found to be present in the plasma of all subjects and was first detected between 2 and 4 h. The PDA was used to calculate the relative MS response of the epoxide compared to lycopene. As the 1,2-epoxide and lycopene have the same chromophore and UV-Vis spectra, they will have equivalent extinction coefficients. The MS response factor was then used to quantitate the ^13^C-labeled lycopene 1,2-epoxide in plasma based on the external calibration curve for ^13^C-labeled lycopene. The ^13^C-lycopene 1,2-epoxide was found to increase linearly over the first 8 h after administration of the ^13^C-lycopene (Figure 4A). Most subjects reached maximum plasma levels between 8 and 9 h after dosing, with concentrations calculated to be 0.82–4.65 nmol/L. This corresponds to 1.71 ± 0.11% of the ^13^C-lycopene detected at those time points. 

Interestingly, on average, there was little change in the ^13^C-epoxide in plasma between 10 and 24 h. However, by 72 h the ^13^C-epoxide had decreased by 70–100% of the maximum concentration and after 28 days was non-detectable in any of the subjects. This time course differs from the ^13^C-lycopene, which continued to increase significantly in plasma between 10 and 24 h and was still quantifiable after 28 days (Figure 4B).

A metabolite corresponding to the unlabeled form of the lycopene 1,2-epoxide was also detected at the same retention time in the plasma of all subjects. The concentrations are low (<1.0 nmol/L), but quantifiable, on the day of dosing (0–10 h) and increased significantly once subjects resumed their moderate lycopene diet (10–20 mg lycopene/d). At 28 days, the unlabeled lycopene 1,2-epoxide was found to be between 1.63 and 11.18 nmol/L in plasma. We have ruled out the lycopene epoxide being an artifact of sample handling based on the changing ratios of the native (unlabeled) epoxide to native lycopene in plasma over the course of the study. The native lycopene epoxide is present in plasma at 0.089 ± 0.027% the concentration of native circulating lycopene on the day of dosing (0–10 h) when unlabeled lycopene was not consumed. However, the native epoxide is present at 0.52 ± 0.16% and 0.50 ± 0.12% the concentration of native lycopene at 72 h and 28 days, respectively, when subjects returned to a moderate lycopene diet.

## 3. Discussion 

In this study, we utilized stable isotope technology to detect and characterize the presence of lycopene epoxides in human biosamples. This effort extends the work of Khachik et al. (1997), reporting the presence of lycopene diols in human serum and hypothesizing that epoxidation is an initial step in the oxidative metabolism of lycopene, followed by enzymatic or acid hydrolysis to generate the diol forms [24,25]. They did not observe the epoxide but invoked it as a metabolic precursor to explain the presence of lycopene diols. Here, we did detect lycopene epoxides consistently in plasma samples from this study using a combination of accurate mass, UV-Vis, and chromatographic behavior, but were unable to detect lycopene diols. This could be an issue of ionization, limit of detection, or stability of these forms. Targeted LC-MS methods should be developed to confirm the presence of lycopene diols along with the epoxide in vivo. 

Lycopene 1,2-epoxide and 5,6-epoxide are known derivatives of lycopene detected in tomatoes and processed tomato products [22,26,27,28,29]. Lycopene epoxides have also been chemically generated with m-chloroperbenzoic acid [20,28] and via autooxidation in low moisture and aqueous model systems [28]. Upon further investigation of our ^13^C-labeled lycopene dose, we found that one of the peaks in the LC chromatogram originally thought to be a minor cis-isomer of lycopene, was actually the 1,2 epoxide. The ^13^C-labeled lycopene 1,2-epoxide was present in the dose at approximately 1% the concentration of lycopene. Additionally, the plasma appearance curve of the ^13^C-labeled lycopene epoxide mimics that of the ^13^C-labeled lycopene over the first 10 h (Figure 4), suggesting that it may be similarly absorbed as ^13^C-labeled lycopene and cleared or metabolized more rapidly. However, alternative explanations may exist. For example, the ^13^C-labeled lycopene 1,2-epoxide in the dose may be very reactive and degrade quickly by hydrolysis during ingestion and digestion, as proposed for many epoxides [24,30]. In that case, the 1,2-epoxide in blood may be part of a dynamic process of epoxidation and diol formation in vivo. The presence of the unlabeled lycopene 1,2 epoxide in plasma the day of dosing, when native lycopene was not consumed, further points to continued metabolism of circulating lycopene through epoxidation. We have ruled out artifactual formation from sample handling as the ratio of the lycopene epoxide to lycopene changes over the course of the study and tracks with lycopene (native and labeled) intake. If the epoxide were being formed during the extraction, we would expect the ratio of the epoxide to lycopene to fluctuate randomly or remain constant.

Future studies with the pure compound are needed to determine whether the lycopene 1,2-epoxide is absorbed from the diet or an early oxidative metabolite of lycopene. Epoxycarotenoids are widely distributed in nature, but xanthophyll epoxides do not appear to be significantly absorbed by humans [31]. The presence of lycopene diols in plasma and the absence of the epoxide forms led Khachick et al. (1998) to hypothesize that the epoxides are not absorbed intact [25]. On the other hand, orally administered dietary and synthetic forms of 5,6-epoxy-β-carotene were found to be bioavailable in humans [32] and have been shown to be biologically active in inducing differentiation of NB4 leukemia cells in vitro [33]. Other epoxy lipids have also been reported to have important biological activity. For example, fatty acid monoepoxides have been reported to have anti-inflammatory properties [34], antinociceptive effects [35], protect cardiovascular function [36], and inhibit angiogenesis and tumorigenesis [37]. However, reactive epoxides may also lead to non-specific macromolecule covalent binding that is deleterious to cells. Therefore, regardless of whether the lycopene epoxide is absorbed or formed in vivo, it is present in human plasma at biologically relevant concentrations and may contribute to some of the bioactivity associated with lycopene and tomatoes. 

In the current study, we were unable to detect ^13^C-labeled or unlabeled apo-lycopenals in plasma at any of the time points. Our group previously reported apo-lycopenals in the plasma of subjects who had been on a high tomato juice dietary intervention for 8 weeks [12]. Despite significant tomato consumption, the concentrations of these oxidation products were quite low in plasma (0.12–0.73 nmol/L). Apo-lycopenals are present in tomato juice, but not our ^13^C-labeled lycopene dose, and therefore, may only accumulate at circulating levels after chronic intake of tomato products. Alternatively, apo-lycopenals may be transient metabolites in vivo, making them challenging to detect from a single dose of ^13^C-lycopene. 

## 4. Materials and Methods 

### 4.1. Clinical Study Design

**Figure 5:**
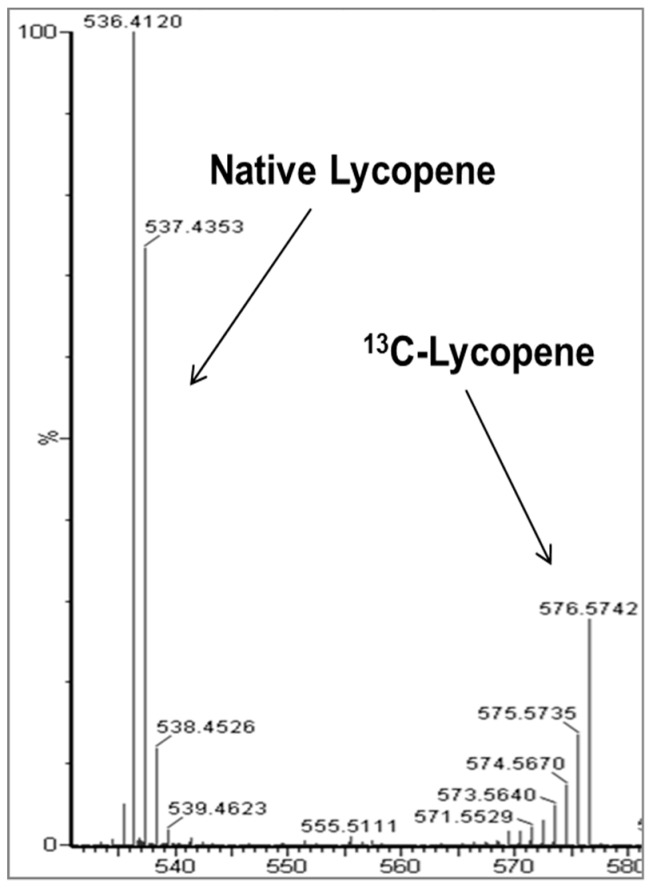
Mass spectrum comparing the isotope distributions for native (unlabeled) lycopene and the ^13^C-labeled lycopene in a representative plasma sample.

Full details of the ^13^C-lycopene dose and pharmacokinetic study, including MS chromatograms and spectra, have been published [18,38]. The ^13^C-lycopene was biosynthesized via tomato cell suspensions cultured with ^13^C-glucose and isolated for human consumption. The lycopene was purified using a crystallization method [38] and the spectroscopic purity was 99% as determined by UV-Vis and no native lycopene was detected in the dose by MS. The resulting ^13^C-lycopene dose was over 91% ^13^C atomic purity and approximately 30% uniformly labeled, giving a unique reverse isotope distribution when analyzed by MS (Figure 5). A total of 8 healthy adults (4 males and 4 females) were administered approximately 10.2 mg of ^13^C-labeled lycopene in olive oil. The oil was added to solubilize the lycopene, which is lipophilic, and because a number of studies have shown that carotenoid bioavailability is much improved with the co-consumption of lipid [39,40,41]. The study was conducted in compliance with the ethical standards of and was approved by The Ohio State University Institutional Review Board (#2009C0104), and written informed consent was obtained from all subjects. Blood was drawn hourly from 0–10 h after dosing and at 1, 3, and 28 days after dosing. Subjects abstained from lycopene containing foods the day of dosing and were instructed to consume a controlled lycopene diet two weeks prior to and four weeks following their visit. Our physiologic compartmental modeling of this study indicated that 23 ± 6% of the ^13^C-lycopene administered was absorbed from the dose [18]. 

### 4.2. Plasma Preparation and LC-MS Analysis

The sample preparation and HPLC-QTOF-MS analysis of the plasma were conducted as previously described [18]. Briefly, plasma was extracted using a mixture of hexane/ethanol/acetone/toluene (10:6:7:7) following protein precipitation with ethanol containing 0.1% butylated hydroxytoluene to prevent oxidation. Extracts were dried under nitrogen and reconstituted in methyl *tert*-butyl ether (MtBE)/methanol (1:1) prior to analysis. All samples were analyzed by HPLC-QTOF-MS the same day they were extracted to minimize potential degradation and oxidation of lycopene and its metabolites. A model 2695 HPLC with a 996 PDA (Waters Corp., Milford, MA, USA) was coupled to a quadrupole time-of-flight (Q-TOF) Premier hybrid mass spectrometer (Micromass UK Ltd., Manchester, UK) via an atmospheric pressure chemical ionization probe operated in negative ion mode. Compounds were separated on a YMC C30 column (3 μm; 4.6 × 250 mm) (Waters Corp.) with a mobile phase gradient consisting of solvent mixtures A (60% methanol, 35% MtBE, and 5% water) and B (78% MtBE, 20% methanol, and 2% water). MS parameters were as follows: corona current, 30 µA; collision energy, 8 eV; cone voltage, 40 V; source block temperature, 110 °C; probe temperature, 600 °C; and desolvation gas flow, 400 L·h^−1^. All MS experiments were run in Enhanced Duty Cycle mode for increased sensitivity with V-optics enabled (7500 mass resolution). MS data were acquired using MassLynx software version 4.1 (Waters Corp.).

### 4.3. Data Mining

The HPLC-QTOF-MS plasma data acquired from this study were mined for new ^13^C-labeled metabolites of lycopene using an untargeted approach. Waters format data files (.raw) were converted to an open data format (.mzXML) using ProteoWizard (version 3.0.10875) [42]. A subset of the data files were then analyzed using Isotope Ratio Outlier Analysis (IROA) ClusterFinder software (IROA Technologies, Bolton, MA, USA), which is a feature finding program designed to search for ^13^C isotope patterns within raw mass spectral data. The resulting features were manually assessed and potential ^13^C-labeled metabolites were evaluated in the full dataset. 

## 5. Conclusions

We have demonstrated that stable isotope labeling is an effective strategy for studying phytochemical metabolites in humans. A lycopene epoxide has been observed in human plasma at biologically relevant concentrations for the first time. As other lipid epoxides have been reported to have biological activity, the lycopene epoxide may contribute to the biological properties associated with lycopene and tomatoes. Follow-up experiments are needed to determine the source of the lycopene epoxide in plasma and to investigate the bioactivity of this oxidation product.

## References

[1] Key T.J., Appleby P.N., Travis R.C., Albanes D., Alberg A.J., Barricarte A., Black A., Boeing H., Bueno-De-Mesquita H.B., Chan J.M. (2015). Carotenoids, retinol, tocopherols, and prostate cancer risk: Pooled analysis of 15 studies. Am. J. Clin. Nutr..

[2] Rowles J.L., Ranard K.M., Applegate C.C., Jeon S., An R., Erdman J.W. (2018). Processed and raw tomato consumption and risk of prostate cancer: A systematic review and dose-response meta-analysis. Prostate Cancer Prostatic Dis..

[3] Tan H.-L., Thomas-Ahner J.M., Moran N.E., Cooperstone J.L., Erdman J.W., Young G.S., Clinton S.K. (2017). β-carotene 9′,10′ oxygenase modulates the anticancer activity of dietary tomato or lycopene on prostate carcinogenesis in the TRAMP model. Cancer Prev. Res..

[4] Boileau T.W.-M., Liao Z., Kim S., Lemeshow S., Erdman J.W., Clinton S.K. (2003). Prostate carcinogenesis in *N*-methyl-*N*-nitrosourea (NMU)-testosterone-treated rats fed tomato powder, lycopene, or energy-restricted diets. J. Natl. Cancer Inst..

[5] Clinton S.K. (1998). Lycopene: Chemistry, biology, and implications for human health and disease. Nutr. Rev..

[6] Eroglu A., Harrison E.H. (2013). Carotenoid metabolism in mammals, including man: Formation, occurrence, and function of apocarotenoids. J. Lipid Res..

[7] Lindshield B.L., Canene-Adams K., Erdman J.W. (2007). Lycopenoids: Are lycopene metabolites bioactive?. Arch. Biochem. Biophys..

[8] Eroglu A., Hruszkewycz D.P., dela Sena C., Narayanasamy S., Riedl K.M., Kopec R.E., Schwartz S.J., Curley R.W., Harrison E.H. (2012). Naturally occurring eccentric cleavage products of provitamin A β-carotene function as antagonists of retinoic acid receptors. J. Biol. Chem..

[9] Eroglu A., Hruszkewycz D.P., Curley R.W., Harrison E.H. (2010). The eccentric cleavage product of β-carotene, β-apo-13-carotenone, functions as an antagonist of RXRα. Arch. Biochem. Biophys..

[10] Caris-Veyrat C., Schmid A., Carail M., Böhm V. (2003). Cleavage products of lycopene produced by in vitro oxidations: Characterization and mechanisms of formation. J. Agric. Food Chem..

[11] Kim S.J., Nara E., Kobayashi H., Terao J., Nagao A. (2001). Formation of cleavage products by autoxidation of lycopene. Lipids.

[12] Kopec R.E., Riedl K.M., Harrison E.H., Curley R.W., Hruszkewycz D.P., Clinton S.K., Schwartz S.J. (2010). Identification and quantification of apo-lycopenals in fruits, vegetables, and human plasma. J. Agric. Food Chem..

[13] Zaripheh S., Boileau T.W.-M., Lila M.A., Erdman J.W. (2003). [^14^C]-lycopene and [^14^C]-labeled polar products are differentially distributed in tissues of F344 rats prefed lycopene. J. Nutr..

[14] Moran N.E., Clinton S.K., Erdman J.W. (2013). Differential bioavailability, clearance, and tissue distribution of the acyclic tomato carotenoids lycopene and phytoene in mongolian gerbils. J. Nutr..

[15] Ross A.B., Vuong L.T., Ruckle J., Synal H.A., Schulze-Konig T., Wertz K., Rumbeli R., Liberman R.G., Skipper P.L., Tannenbaum S.R. (2011). Lycopene bioavailability and metabolism in humans: An accelerator mass spectrometry study. Am. J. Clin. Nutr..

[16] Fleshman M.K., Riedl K.M., Novotny J.A., Schwartz S.J., Harrison E.H. (2012). An LC/MS method for d8-β-carotene and d4-retinyl esters: β-carotene absorption and its conversion to vitamin A in humans. J. Lipid Res..

[17] Kurilich A.C., Britz S.J., Clevidence B.A., Novotny J.A. (2003). Isotopic labeling and LC-APCI-MS quantification for investigating absorption of carotenoids and phylloquinone from kale (*Brassica oleracea*). J. Agric. Food Chem..

[18] Moran N.E., Cichon M.J., Riedl K.M., Grainger E.M., Schwartz S.J., Novotny J.A., Erdman J.W., Clinton S.K. (2015). Compartmental and noncompartmental modeling of ^13^C-lycopene absorption, isomerization, and distribution kinetics in healthy adults. Am. J. Clin. Nutr..

[19] Moran N.E., Novotny J.A., Cichon M.J., Riedl K.M., Rogers R.B., Grainger E.M., Schwartz S.J., Erdman J.W., Clinton S.K. (2016). Absorption and distribution kinetics of the ^13^C-labeled tomato carotenoid phytoene in healthy adults. J. Nutr..

[20] Khachik F., Steck A., Niggli U.A., Pfander H. (1998). Partial synthesis and structural elucidation of the oxidative metabolites of lycopene identified in tomato paste, tomato juice, and human serum. J. Agric. Food Chem..

[21] Mercadante A.Z., Egeland E.S., Britton G., Liaaen-Jensen S., Pfander H. (2004). Carotenoids Handbook.

[22] Khachik F., Goli M.B., Beecher G.R., Holden J., Lusby W.R., Tenorio M.D., Barrera M.R. (1992). Effect of food preparation on qualitative and quantitative distribution of major carotenoid constituents of tomatoes and several green vegetables. J. Agric. Food Chem..

[23] Eugster C.H., Britton G., Liaaen-Jensen S., Pfander H. (1995). Chemical Derivatization: Microscale Tests for the Presence of Common Functional Groups in Carotenoids. Carotenoids Volume 1A: Isolation and Analysis.

[24] Khachik F., Spangler C.J., Smith J.C., Canfield L.M., Steck A., Pfander H. (1997). Identification, quantification, and relative concentrations of carotenoids and their metabolites in human milk and serum. Anal. Chem..

[25] Khachik F., Pfander H., Traber B. (1998). Proposed mechanisms for the formation of synthetic and naturally occurring metabolites of lycopene in tomato products and human serum. J. Agric. Food Chem..

[26] Britton G., Goodwin T.W. (1975). Carotene epoxides from the Delta tomato mutant. Phytochemistry.

[27] Tonucci L.H., Holden J.M., Beecher G.R., Khachik F., Davis C.S., Mulokozi G. (1995). Carotenoid content of thermally processed tomato-based food products. J. Agric. Food Chem..

[28] Rodriguez E.B., Rodriguez-Amaya D.B. (2009). Lycopene epoxides and apo-lycopenals formed by chemical reactions and autoxidation in model systems and processed foods. J. Food Sci..

[29] Cichon M.J., Riedl K.M., Schwartz S.J. (2017). A metabolomic evaluation of the phytochemical composition of tomato juices being used in human clinical trials. Food Chem..

[30] Newman J.W., Morisseau C., Hammock B.D. (2005). Epoxide hydrolases: Their roles and interactions with lipid metabolism. Prog. Lipid Res..

[31] Barua A.B., Olson J.A. (2001). Xanthophyll epoxides, unlike beta-carotene monoepoxides, are not detectibly absorbed by humans. J. Nutr..

[32] Barua A.B. (1999). Intestinal absorption of epoxy-beta-carotenes by humans. Biochem. J..

[33] Duitsman P.K., Barua A.B., Becker B., Olson J.A. (1999). Effects of epoxycarotenoids, beta-carotene, and retinoic acid on the differentiation and viability of the leukemia cell line NB4 in vitro. Int. J. Vitam. Nutr. Res..

[34] Node K., Huo Y., Ruan X., Yang B., Spiecker M., Ley K., Zeldin D.C., Liao J.K. (1999). Anti-inflammatory properties of cytochrome P450 epoxygenase-derived eicosanoids. Science.

[35] Wagner K., Vito S., Inceoglu B., Hammock B.D. (2014). The role of long chain fatty acids and their epoxide metabolites in nociceptive signaling. Prostaglandins Other Lipid Mediat..

[36] Gauthier K.M., Yang W., Gross G.J., Campbell W.B. (2007). Roles of epoxyeicosatrienoic acids in vascular regulation and cardiac preconditioning. J. Cardiovasc. Pharmacol..

[37] Zhang G., Panigrahy D., Mahakian L.M., Yang J., Liu J.-Y., Stephen Lee K.S., Wettersten H.I., Ulu A., Hu X., Tam S. (2013). Epoxy metabolites of docosahexaenoic acid (DHA) inhibit angiogenesis, tumor growth, and metastasis. Proc. Natl. Acad. Sci. USA.

[38] Moran N.E., Rogers R.B., Lu C.-H., Conlon L.E., Lila M.A., Clinton S.K., Erdman J.W. (2013). Biosynthesis of highly enriched ^13^C-lycopene for human metabolic studies using repeated batch tomato cell culturing with ^13^C-glucose. Food Chem..

[39] Goltz S.R., Campbell W.W., Chitchumroonchokchai C., Failla M.L., Ferruzzi M.G. (2012). Meal triacylglycerol profile modulates postprandial absorption of carotenoids in humans. Mol. Nutr. Food Res..

[40] Unlu N.Z., Bohn T., Clinton S.K., Schwartz S.J. (2005). Carotenoid absorption from salad and salsa by humans is enhanced by the addition of avocado or avocado oil. J. Nutr..

[41] White W.S., Zhou Y., Crane A., Dixon P., Quadt F., Flendrig L.M. (2017). Modeling the dose effects of soybean oil in salad dressing on carotenoid and fat-soluble vitamin bioavailability in salad vegetables. Am. J. Clin. Nutr..

[42] Chambers M.C., Maclean B., Burke R., Amodei D., Ruderman D.L., Neumann S., Gatto L., Fischer B., Pratt B., Egertson J. (2012). A cross-platform toolkit for mass spectrometry and proteomics. Nat. Biotechnol..

